# Essence of Chicken Supplementation Alters Brain and Blood Metabolite Signatures in Sleep-Deprived Mice

**DOI:** 10.3390/metabo15090577

**Published:** 2025-08-29

**Authors:** Yu Long, Zhaorong Wang, Xinyang Hu, Sisi Wang, Liujie Zheng, Zhengwei Fu, Yinhua Ni

**Affiliations:** 1 College of Pharmacy, Zhejiang University of Technology, Hangzhou 310032, China; 2386603@zjut.edu.cn; 2 College of Biotechnology and Bioengineering, Zhejiang University of Technology, Hangzhou 310032, China; 211122050010@zjut.edu.cn (Z.W.); 221123050209@zjut.edu.cn (X.H.); 221123050193@zjut.edu.cn (S.W.); 2112005011@zjut.edu.cn (L.Z.); azwfu@zjut.edu.cn (Z.F.)

**Keywords:** essence of chicken, sleep deprivation, bioactives, metabolomics, neurotransmitters

## Abstract

**Background**: Essence of chicken (EC) has been found to improve brain function, increase short-term working memory, and reduce fatigue. However, the specific bioactives after EC consumption remain unknown, and the effect of EC on sleep deprivation (SD) is also elusive. The aim of the present study is to clarify the metabolic changes induced by EC supplementation in the serum and brain and identify characteristic bioactive metabolites significantly altered after EC consumption. **Methods**: Firstly, a kinetic analysis of EC consumption was performed to determine the time-sequential change in serum and brain metabolites in mice using gas chromatography coupled with mass spectrometry (GC/MS). Next, the impact of EC on the metabolic signatures in an acute SD mouse model was assessed. **Results**: Based on the results of the kinetic study, myristoleic acid and L-tyrosine were significantly increased in the serum, whereas gentisic acid was significantly increased in the brain after the administration of EC. In addition, EC administration differentially modulated SD-induced alterations in gene expression across brain regions of acute sleep-deprived mice, ameliorated abnormal neurotransmitters, and increased several specific metabolites in the serum. **Conclusions**: These results suggest that EC might be an effective nutritional supplement to alleviate SD-induced physiological changes.

## 1. Introduction

Sleep is an important physiological process that promotes central nervous system (CNS) metabolism, strengthens memory, and regulates mood [1,2]. With the increasing demands of modern life and external pressure, sleep time and quality are declining rapidly. A study of more than 2000 subjects reported prevalence rates of 32.1% for general sleep disturbance, 43.2% for insufficient sleep, 8.2% for insomnia, and 5.3% for circadian sleep disturbance [3]. People sleep less now compared to in the past, with approximately 30% of adults sleeping less than 7 h [4]. According to reports, Asian adults slept for less than 6.5 h per night in 2023 [5], far below the 7–9 h standard recommended in Europe and by experts [6]. Lack of sleep disrupts metabolism and increases the risk of immune system disorders and cognitive impairment [7,8,9]. Sang et al. found that excessive efflux of the sleep mediator prostaglandin D2 across the blood–brain barrier (BBB) during sleep deprivation (SD) causes a cytokine-storm-like syndrome that induces multiple organ dysfunction syndrome and death [7]. In addition, there is substantial evidence that long-term sleep restriction, or sleep fragmentation, reduces dentate gyrus neurogenesis in adult animals, impairs hippocampal synaptic plasticity, and undermines hippocampal-dependent memory consolidation, thus affecting cognitive functions [8,9].

Sleep disorders are not only associated with decreased quality of life and productivity [10] but also cause severe damage to multiple systems and organs of the body, including the CNS and the cardiovascular, digestive, respiratory, endocrine, immune, and reproductive systems [11]. SD refers to sleep disorders caused by reduced rest time and quality due to environmental or personal factors [12]. Studies have revealed the impact of SD on the CNS, such as cognitive impairment, as well as the deterioration of neurological, psychiatric, and emotional disorders [13,14,15]. For those who experience SD, the rational and moderate use of some psychostimulants might be timely and warranted [16]. However, these drugs often come with several side effects, including excessive sleepiness, cognitive impairment, nocturnal wandering, restlessness, and balance problems, which limit their clinical use [17]. Therefore, there is an urgent need to clarify the potential mechanisms of SD and develop effective treatment methods. A recent study found that fructus gardeniae alleviates anxiety-like behaviors induced by SD, and 15 potential biomarkers were identified in the hippocampus using metabolomic techniques [18]. These biomarkers were used to reduce SD-induced neuroinflammation and improve sleep disorders [18], highlighting the validity and feasibility of dietary nutrition intervention in ameliorating sleep disorders. In contrast to such phytochemical-based approaches, our study presents the first metabolic profiling of animal meat-derived essence of chicken (EC) supplementation in the context of SD, offering novel mechanistic insights into the beneficial effects of EC.

Total SD is the most commonly used sleep manipulation technique in rodents, and many SD paradigms have been developed in mice, including gentle handling, a moving treadmill, the roller method, multiple platforms over water, and curling prevention by water [7,19,20]. These environmental disturbances induce SD depending on the modeling time and cause the animals to exhibit insomnia symptoms characterized by reduced sleep duration [21]. Yu et al. investigated the effect of acute SD on nitroglycerin-induced pain sensitivity by continuously providing small paper balls, plastic balls, and gauze to mice cages and monitoring them to stay awake for 6 h [22]. This method has the advantages of low stimulation intensity, small disturbance to animals, no continuous pressure, and easy implementation, which makes it suitable for modeling acute SD [22]. Based on the above, we used the gentle handling method to induce 6 h of acute SD in mice to induce sleep disorder symptoms in the present study.

EC is a liquid nutritional supplement commonly used in Southeast Asia and traditional Chinese medicine, with multiple health benefits and remedies [23]. EC is a functional food prepared by boiling chicken in water under high pressure and temperature to convert long-chain proteins into short-chain proteins or peptides, thereby making it rich in proteins, trace elements, carnosine, creatinine, and amino acids [24,25]. It has been recognized that EC relieves physical and mental stress, increases attention, assists in correcting biological clock disorder, and promotes metabolism [26]. For example, Yamano et al. demonstrated that daily EC supplementation relieved mental fatigue in healthy male volunteers [27]. Studies have shown that carnosine and anserine in EC possess antioxidant activity, which can protect brain cells from oxidative damage [28]. In addition, glutamate, one of the key components in EC, plays a central role in synaptic plasticity, which is involved in the mechanism of long-term potentiation through the NMDA receptor, thereby affecting cognitive function [29]. Studies have found that the levels of certain amino acids in plasma, such as tryptophan, serotonin, and taurine, significantly increase during SD, and these metabolites may alleviate the negative impacts of SD by participating in the synthesis of neurotransmitters or regulating the function of nerve cells [30]. Therefore, metabolites play an important role in the regulation of brain function and the alleviation of physiological changes caused by SD. However, the impact of EC on SD and the metabolomic changes after EC consumption remain elusive.

Herein, we hypothesized that EC might alleviate SD-related metabolic changes by increasing specific metabolites and bioactives in mice. To test this hypothesis, we first used metabolomics analysis based on liquid chromatography–mass spectrometry (LC-MS) to identify the changes in serum and brain metabolic profiles of mice after supplementing with EC at different time points. Following this, the changes in characteristic metabolites and metabolic pathways in the serum and brain of SD mice supplemented with EC were determined.

## 2. Materials and Methods

### 2.1. Dosage Information

The EC used in this study was prepared from chicken meat, as described previously [31]. The daily dose of EC for humans is 5.4 g powder/day for a 60 kg human, which was converted to 1107 mg/kg body weight (BW)/day in mice (defined as 1× EC dose) using body surface area (BSA) normalization (human-to-mouse Km ratio = 12.3) [32]. This dosage is also consistent with our prior studies [33]. The EC powder was dissolved in saline, and the mice were orally gavaged with the EC mixture or an equal volume of saline.

### 2.2. Animals and Experimental Design

Seven-week-old male C57BL/6J mice were obtained from the China National Laboratory Animal Resource Center (Shanghai, China). Mice were kept in a clean animal facility with a constant temperature (22–25 °C) and controlled lighting (12/12 h dark/light cycle) for at least one week for acclimatization. Food and water were given ad libitum. To first measure the concentrations of specific EC bioactives in the blood and brain, a kinetic study was performed. Mice were fasted overnight and divided into 3 groups: (1) control group, mice were gavaged with saline; (2) 1× EC group, mice were gavaged with 1107 mg/kg EC; and (3) 2× EC group, mice were gavaged with 2214 mg/kg EC. At the baseline and after 15, 30, 60, and 120 min of EC administration, the mice were anesthetized with 4% isoflurane in 100% oxygen (*n* = 6 per group per time point). The blood and brain tissues were collected for untargeted metabolomics analysis.

Next, to determine the effects of EC on plasma metabolome and brain function markers in SD mice, mice were divided into 5 groups as follows: (1) control group, mice were orally administrated saline, *n* = 8; (2) control + EC group, mice were orally gavaged with 1107 mg/kg EC, *n* = 8; (3) SD group, mice were sleep-deprived for 6 h and then orally gavaged with saline, *n* = 8; (4) SD + 1× EC group, mice were sleep-deprived for 6 h and then orally gavaged with 1107 mg/kg EC, *n* = 8; and (5) SD + 2× EC group, mice were sleep-deprived for 6 h and then orally gavaged with 2214 mg/kg EC, *n* = 8. After 30 min of EC gavage, the mice were anesthetized with 4% isoflurane in 100% oxygen. The tissues were collected for further analysis. All experiments were approved by the laboratory animals ethical committee of Zhejiang University of Technology and followed the NIH guide for laboratory animals (NIH Publication No. 85-23, revised 1996) for the care and use of animals (Ethical No. MGS20230315030).

### 2.3. Sleep Deprivation Procedure

SD was initiated at the beginning of the light cycle, and the procedure was conducted using a 6 h mild conditioning protocol, as described previously [34]. Specifically, mice were kept awake from 8:00 AM to 2:00 PM by introducing paper tubes, balled paper towels, and plastic balls into the cage. The cage was knocked on if the introduced material did not keep them awake. Non-sleep-deprived mice were placed in a room by themselves with lighting and humidity the same as the SD room and left undisturbed for 6 h.

### 2.4. Quantitative Real-Time Polymerase Chain Reaction

Total RNA from mouse hippocampus, cortex samples, and pineal gland (*n* = 5–8 per group) was extracted using the Tiozol reagent (Vazyme, Nanjing, China), and RNA concentration was assessed using a Nano-3000 instrument (Aosens, Hangzhou, China). The cDNA was synthesized using a high-capacity cDNA reverse transcriptase kit (Tsingke Co., Ltd., Beijing, China), and qPCR was performed using SYBR Green Real-time PCR Master Mix (Tsingke Co., Ltd., Beijing, China) on a CFX Connect Real-Time PCR System788BR09833 (Bio-rad, Hercules, CA, USA), as described previously [35]. β-actin transcription was performed for its use as a housekeeping gene for data standardization. The primer sequences are listed in Table S1.

### 2.5. Enzyme-Linked Immunoassay (ELISA) Assay

The whole blood samples from the mice were centrifuged at 1000 rpm for 15 min, and the supernatants were collected for the experiment. The levels of 5-hydroxytryptamine (5-HT) in the serum (*n* = 4–6 per group) were determined using the corresponding ELISA kits from Cusabio Technology Co., Ltd. (Wuhan, China) according to the manufacturer’s instructions.

### 2.6. Untargeted Metabolic Profiling

Mouse serum and brain samples (*n* =5 per group) were swirled in a centrifuge tube containing 400 μL of methanol for 1 min. Following centrifugation at 12,000 rpm for 10 min at 4 °C, the supernatant was collected and then resuspended in 80% methanol containing 2-chloro-L-phenylalanine. UHPLC-MS/MS analysis was conducted using a Vanquish UHPLC system (Thermo Fisher, Waltham, MA, USA) coupled with an Orbitrap Exploris 120 mass spectrometer (Thermo Fisher, USA) for the assessment of serum and brain samples [36]. The chromatographic conditions were as follows: column (2.1 mm × 100 mm, 1.8 μm), temperature set to 40 °C, flow rate at 0.300 mL/min, mobile phase A consisting of water with 0.05% ammonium, and mobile phase B consisting of acetonitrile. The injection volume was 5 μL, and the auto-sampler temperature was maintained at 4 °C. The mass spectrometry parameters were as follows: sheath gas flow rate set to 45 Arb, auxiliary gas flow rate at 15 Arb, capillary temperature at 350 °C, and spray voltage set to 3.0 kV for positive ion mode and 3.2 kV for negative ion mode.

### 2.7. Targeted Metabolic Profiling

Mouse serum (*n* = 5 per group) was extracted in 10% formic acid in methanol/water (1:1, *v*/*v*), centrifuged at 12,000 rpm and 4 °C for 5 min, and then the supernatant was collected. Targeted metabolomics for 15 neurotransmitters, including kynurenic acid, kynurenine, noradrenaline, glutamine, picolinic acid, levodopa, tryptophan, tyrosine, glutamate, 4-aminobutyric acid, histidine, acetylcholine chloride, histamine, 5-hydroxytryptamine, and 5-hydroxyindole acetic acid, was performed using LC/MS. The LC analysis was performed on an EXion LC liquid chromatograph (AB SCIEX, Framingham, MA, USA). Mass spectrometric detection of metabolites was performed on AB6500+ (AB SCIEX, USA). Data acquisition and processing were performed with Analyst software (version 1.6).

### 2.8. Metabolomic Data Analysis

The qualitative and quantitative results of the metabolites were normalized using Z-score normalization to eliminate differences in scale and magnitude between different samples, facilitating subsequent statistical analyses. Stringent selection criteria were used to identify significantly altered metabolites. Initially, preliminary screening of the metabolite data using principal coordinate analysis (PCA) was performed to assess the overall differences between samples. Subsequently, univariate statistical analyses, such as *t*-tests and ANOVA, were utilized to determine significantly changed metabolites, setting the significance level at *p*-values < 0.05. Moreover, the fold changes in the metabolites were calculated, and thresholds of ≥1.5 or ≤0.5 were established to further filter out metabolites with biological significance, which were then visualized in the form of a volcano plot. Subsequently, pathway analysis of these significantly different metabolites was conducted using the Kyoto Encyclopedia of Genes and Genomes (KEGG) pathway database, with the results presented in a bubble plot. The primary multivariate data analyses were conducted using R software (version 4.0.3), and the differential metabolites underwent pathway analysis through MetaboAnalyst (version 5.0).

### 2.9. Statistical Analysis

All data are given as the mean ± standard error of the mean (SEM). Power analysis showed that *n* = 3 per group was sufficient to detect differences, according to the previous study [37]. Comparisons between different groups were analyzed via one-way analysis of variance (ANOVA) followed by the Tukey–Kramer test. *p*-values < 0.05 or 0.01 were considered statistically significant.

## 3. Results

### 3.1. Effect of EC on Serum Metabolomics Based on the Kinetic Study

**Figure 1:**
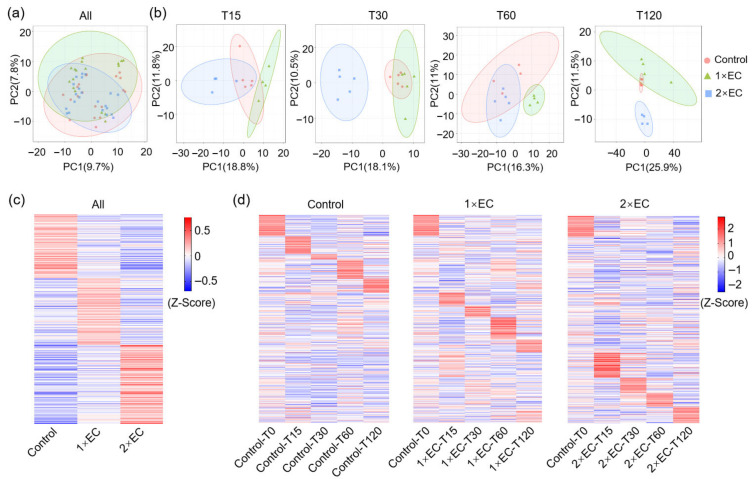
EC administration altered metabolites in the serum in a time-dependent manner. (**a**) PCA of metabolites in three groups with all time points. (**b**) PCA of metabolites in three groups at different time points. (**c**) Z-score-derived heatmap of metabolites in three groups with all time points. (**d**) Z-score-derived heatmap of metabolites in the Control, 1× EC, and 2× EC group with time-dependent change. The sequence of metabolites was the same and arranged according to the Control group.

The specific EC bioactives were determined through untargeted LC/MS metabolomics, and principal coordinate analysis (PCA) of all time points in the control, 1× EC, and 2× EC groups revealed slight discrimination among the three groups (Figure 1a). The separations between the control and EC groups were clearer when analyzed according to the indicated time points (Figure 1b). LC/MS identified over 400 metabolites in the serum, and the heatmap of metabolites in all three groups at all time points showed marked differences between the control and EC groups (Figure 1c). The time-dependent change in metabolites in the three groups was further analyzed, and there was a clear variation in the metabolites within the control group (Figure 1d). EC administration caused a clear time-dependent change in the levels of specific metabolites, with a more obvious change at T15 and T30 (Figure 1d).

KEGG pathway analysis of the TOP20 functions of metabolites in the serum revealed that EC administration caused a marked alteration in the enriched pathways at T15 and T30 (Figure S1). Though the dose- and time-dependent effects of EC on KEGG pathways varied, some common pathways were affected by 1× EC and 2× EC at T15 and T30 (Figure S1). For example, functions related to protein digestion and absorption; phenylalanine, tyrosine, and tryptophan biosynthesis; central carbon metabolism in cancer; ATP-binding cassette (ABC) transporters; biosynthesis of various secondary metabolites-part 3; cholesterol metabolism; secondary bile acid biosynthesis; and taurine and hypotaurine metabolism were all significantly changed by EC at T15 (Figure S1a,b). Functions related to central carbon metabolism in cancer; protein digestion and absorption, linoleic acid metabolism; mineral absorption; aminoacyl-tRNA biosynthesis; phenylalanine, tyrosine, and tryptophan biosynthesis; glucagon signaling pathway; and cocaine addiction were changed by EC at T30 (Figure S1c,d). The common pathways affected by two doses of EC at both time points were central carbon metabolism in cancer and phenylalanine, tyrosine, and tryptophan biosynthesis (Figure S1).

### 3.2. Effect of EC on the Serum Metabolite Profiles of Mice at Different Time Points

**Figure 2:**
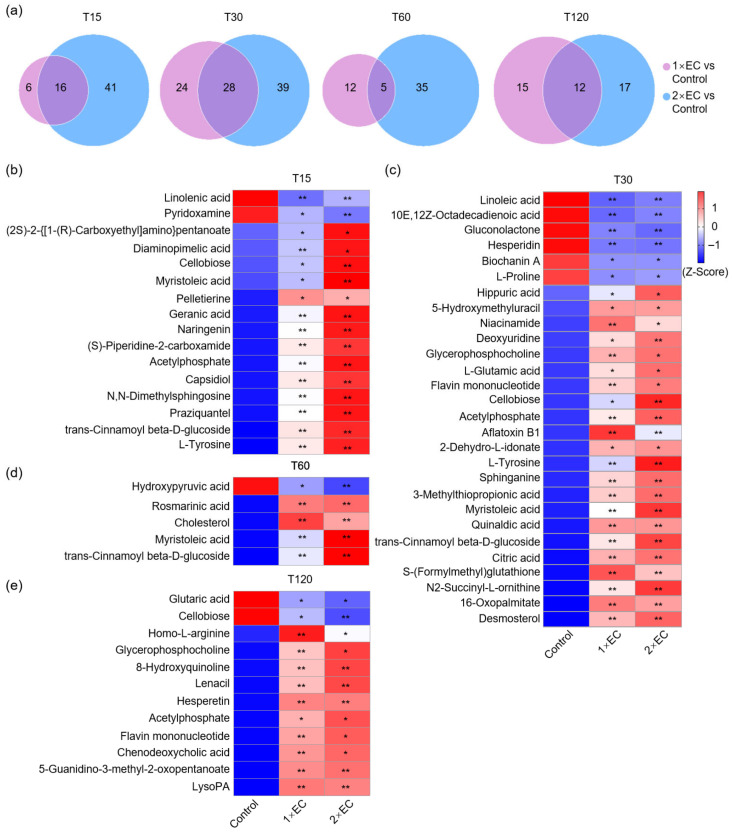
EC significantly affected the serum metabolite levels at different time points. (**a**) Venn diagram of the number of metabolites altered by EC at different time points. Fold-change ≥ 1.5, or ≤0.5, *p* < 0.05. (**b**–**e**) Heatmap of common metabolites altered by 1× EC and 2× EC compared to the Control group at T15 (**b**), T30 (**c**), T60 (**d**), and T120 (**e**). * *p* < 0.05, and ** *p* < 0.01 vs. the Control group.

Next, to find the common metabolites affected by 1× EC and 2× EC, Venn diagram analyses were performed. There were 22 and 57 metabolites significantly changed by 1× EC and 2× EC, respectively, and 16 commonly affected metabolites at T15 compared to the control group (Figure 2a). The numbers of common metabolites were 28, 5, and 12 at T30, T60, and T120, respectively (Figure 2a). The heatmap of these common metabolites at T15 showed that the levels of linolenic acid and pyridoxamine were significantly decreased after EC administration, while the levels of (2S)-2-{[1-(R)-carboxyethyl]amino}pentanoate, myristoleic acid, cellobiose, diaminopimelic acid, geranic acid, acetylphosphate, N,N-dimethylsphingosine, naringenin, praziquantel, L-tyrosine, (S)-piperidine-2-carboxamide, capsidiol, trans-cinnamoyl beta-D-glucoside (a sharp increase presumably resulting from a dietary metabolite originating from the corncob bedding consumed by the mice), and pelletierine were markedly increased by EC (Figure 2b). Similarly, the levels of biochanin A, hesperidin, L-proline, gluconolactone, linoleic acid, and 10E,12Z-octadecadienoic acid were decreased, and the levels of cellobiose, L-tyrosine, hippuric acid, aflatoxin B1, myristoleic acid, trans-cinnamoyl beta-D-glucoside, N2-succinyl-L-ornithine, acetylphosphate, L-glutamic acid, niacinamide, deoxyuridine, sphinganine, S-(formylmethyl)glutathione, 3-methylthiopropionic acid, flavin mononucleotide, desmosterol, glycerophosphocholine, citric acid, 2-dehydro-L-idonate, 16-oxopalmitate, quinaldic acid, and 5-hydroxymethyluracil were increased by both doses of EC treatment at T30 (Figure 2c). The level of hydroxypyruvic acid was decreased by EC at T60, and the levels of myristoleic acid, trans-cinnamoyl beta-D-glucoside, cholesterol, and rosmarinic acid were increased by EC (Figure 2d). Finally, the levels of glutaric acid and cellobiose were decreased, and the levels of homo-L-arginine, glycerophosphocholine, 8-hydroxyquinoline, lenacil, acetylphosphate, flavin mononucleotide, chenodeoxycholic acid, 5-guanidino-3-methyl-2-oxopentanoate, lysoPA, and hesperetin were increased by EC at T120 (Figure 2e).

To also find the bioactives that might be critical for EC consumption, the metabolites significantly increased by EC were visualized using line graphs (Figure S2). The levels of metabolites showed a clear time-dependent change, with a peak at T15 or T30 for most of them (Figure S2). Among these metabolites, the most obviously changed were myristoleic acid, L-tyrosine, and trans-cinnamoyl beta-D-glucosid, with the peak at T15, and the levels increased over 10-fold (Figure S2). In addition, the levels of geranic acid, acetylphosphate, N,N-dimethylsphingosine, naringenin, praziquantel, capsidiol, hippuric acid, N2-succinyl-L-ornithine, 3-methylthiopropionic acid, flavin mononucleotide, and 16-oxopalmitate were also increased over 2-fold with a peak time at T15 or T30 (Figure S2).

### 3.3. Effect of EC on Brain Metabolomics Based on Kinetic Study

**Figure 3:**
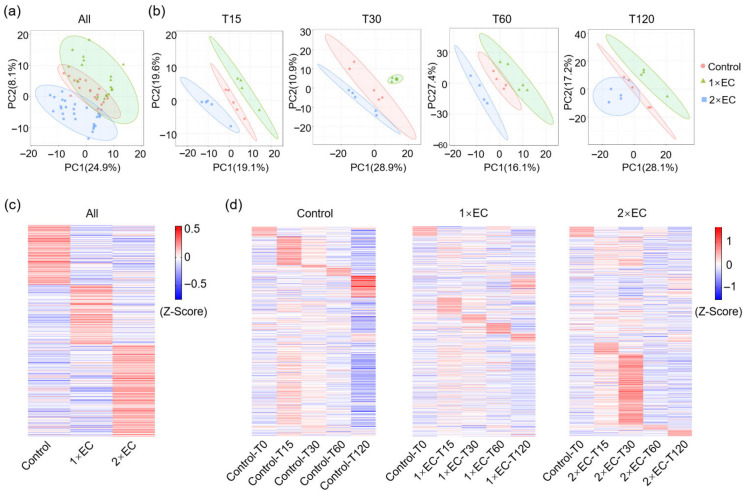
EC administration changed metabolites in the brain in a time-dependent manner. (**a**) PCA of metabolites in three groups with all time points. (**b**) PCA of metabolites in three groups at different time points. (**c**) Z-score-derived heatmap of metabolites in three groups with all time points. (**d**) Z-score-derived heatmap of metabolites in the Control, 1× EC, and 2× EC groups with time-dependent change. The sequence of metabolites was the same and arranged according to the Control group.

Next, the effect of EC on brain metabolites was determined, and PCA of all time points in the control, 1× EC, and 2× EC groups revealed a clear discrimination among the three (Figure 3a). The differences between the control and EC groups were clearly separated when analyzed by the indicated time points (Figure 3b). The heatmaps of metabolites in the three groups at all time points showed marked differences between the control and EC groups (Figure 3c). The time-dependent change in metabolites in the three groups was further analyzed, and, similarly, a clear variation was identified in the metabolites within the control group (Figure 3d). EC administration caused a marked increase in specific metabolites over time, and the most obvious change in metabolites was found at T30 (Figure 3d). Consistent with serum metabolomics, the functions of metabolites in the brain were also analyzed at 15 and 30 min (Figure S3). KEGG pathway analysis of the TOP20 functions of metabolites in the brain revealed that EC administration caused a marked alteration in the enriched pathways at T15 and T30, and several common pathways were affected by 1× EC and 2× EC at both time points, for example, alanine, aspartate, glutamate metabolism, central carbon metabolism in cancer, and protein digestion and absorption (Figure S3).

### 3.4. Effect of EC on the Brain Metabolite Profiles of Mice at Different Time Points

**Figure 4:**
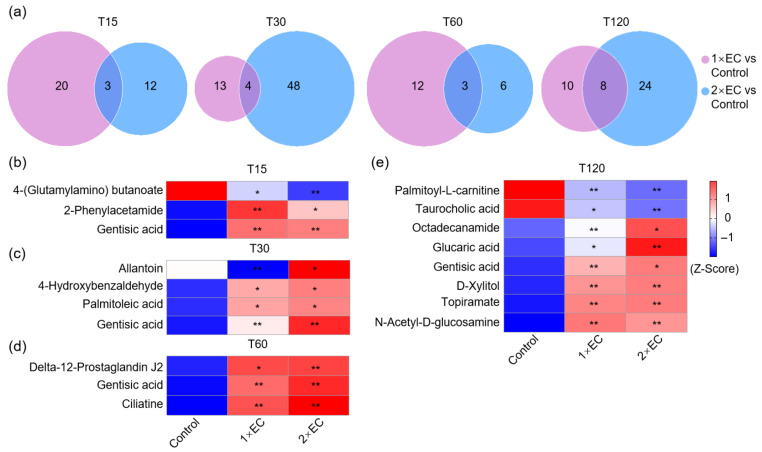
EC significantly affected the brain metabolite levels at different time points. (**a**) Venn diagram of the number of metabolites altered by EC at different time points. Fold-change ≥ 1.5, or ≤0.5, *p* < 0.05. (**b**–**e**) Heatmap of common metabolites altered by 1× EC and 2× EC compared to the Control group at T15 (**b**), T30 (**c**), T60 (**d**), and T120 (**e**). * *p* < 0.05, and ** *p* < 0.01 vs. the Control group.

Next, the Venn diagram revealed that 23 and 15 metabolites were significantly changed by 1× EC and 2× EC, respectively, and there were only 3 commonly affected metabolites at T15 compared to the control group (Figure 4a). The numbers of common metabolites were 4, 3, and 8 at T30, T60, and T120, respectively (Figure 4a). The heatmap of these common metabolites at T15 showed that the level of 4-(glutamylamino) butanoate was significantly decreased after EC administration, while the levels of 2-phenylacetamide and gentisic acid were markedly increased by EC (Figure 4b). Similarly, the levels of allantoin, gentisic acid, 4-hydroxybenzaldehyde, palmitoleic acid, ciliatine, gentisic acid, and delta-12-prostaglandin J2 were increased by both doses of EC treatment at T30 and T60 (Figure 4c,d). The levels of palmitoyl-L-carnitine and taurocholic acid were decreased by EC at T120, and the levels of gentisic acid, octadecanamide, glucaric acid, N-acetyl-D-glucosamine, D-xylitol, and topiramate were increased by EC (Figure 4e).

The line graphs showed that the levels of metabolites displayed a clear time-dependent change, with most displaying a peak mainly at T30 (Figure S4). Among these metabolites, the most obviously changed metabolite was gentisic acid, with a significant increase of over 10-fold and a peak time at T30 (Figure S4). Though the level of allantoin increased over 5-fold in the 2× EC group, the dose-dependent effect was unclear (Figure S4).

### 3.5. Effect of EC on Serum Neurotransmitter Levels in the SD Mice

Next, the effect of EC on SD was determined. The mRNA expression of the markers related to SD in the cerebral cortex and hippocampus was first analyzed via RT-qPCR. In the cerebral cortex and hippocampus, SD caused a significant increase in the expression of these genes compared to the control group, while EC downregulated the expression of these genes in a dose-dependent manner (Figure S5). However, 1× EC administration alone did not affect the expression of these genes in the cortex of control mice (Figure S5a,b). Next, the mRNA expression of melatonin formation marker, arylalkylamine N-acetyltransferase (*Aanat*), and 5-HT metabolism marker, monoamine oxidase type B (*Mao-b*) and tryptophan hydroxylase 2 (*Tph2*), in the pineal gland was determined. SD resulted in a significant decrease in the expression of *Aanat* in the pineal gland, and EC treatment upregulated the expression of this gene (Figure S5c). On the other hand, the expression of *Mao-b* was increased by SD and decreased by EC in the pineal gland. In contrast, the expression of *Tph2* was downregulated by SD and reversed by EC (Figure S5c).

**Figure 5:**
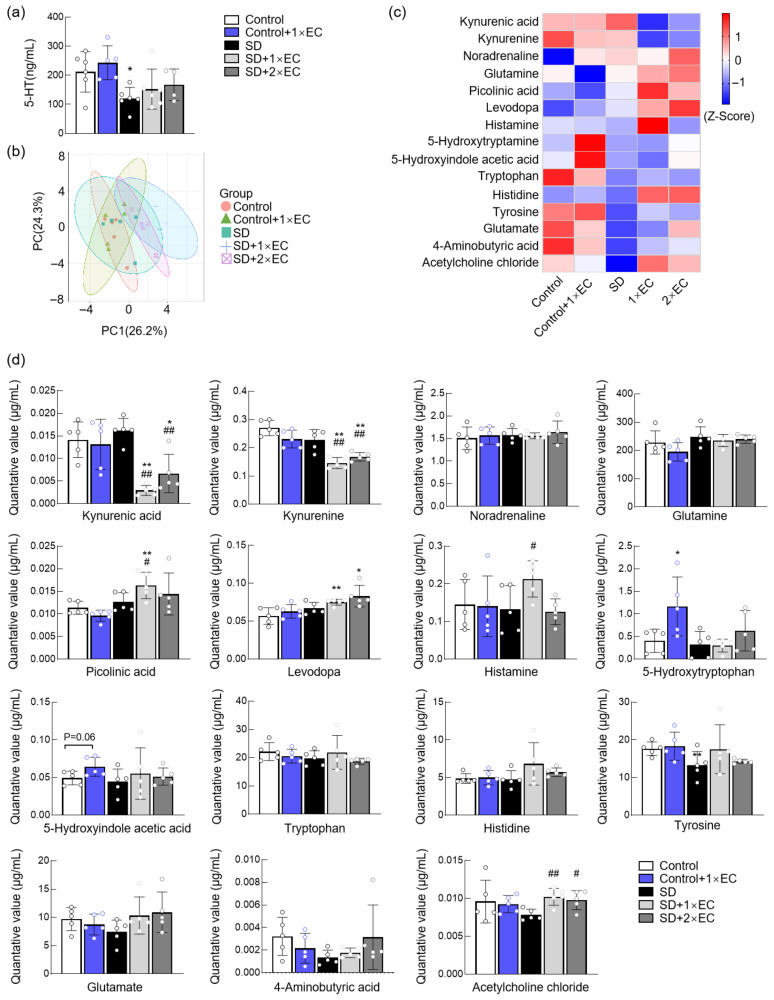
EC administration improved abnormal neurotransmitter levels in the serum of SD mice. (**a**) Serum 5-HT levels. (**b**) PCA of neurotransmitters by targeted metabolomics. (**c**) Heatmap of detected neurotransmitters in the serum by targeted metabolomics. (**d**) Quantitative levels of neurotransmitters in the serum. * *p* < 0.05, and ** *p* < 0.01 vs. the Control group, # *p* < 0.05, and ## *p* < 0.01 vs. the SD group.

To first determine whether EC administration could affect the levels of neurotransmitters, ELISA analysis of serum 5-HT level, as one of the most abundant neurotransmitters, was first conducted. SD caused a marked decrease in the serum 5-HT level, which tended to be increased by EC (Figure 5a). To fully clarify the effect of EC on neurotransmitters, targeted metabolomics for neurotransmitters was performed, and a total of 15 neurotransmitters were detected and identified. PCA revealed a slight separation among different groups (Figure 5b). The heatmap of these 15 neurotransmitters showed that SD tended to increase the level of several neurotransmitters, such as kynurenic acid, noradrenaline, glutamine, picolinic acid, and levodopa, and also tended to decrease the level of tryptophan, tyrosine, glutamate, 4-aminobutyric acid, and acetylcholine chloride; EC treatment reversed the changes in these neurotransmitters to some extent (Figure 5c). The quantitative results further show that both doses of EC significantly reduced the levels of kynurenic acid and kynurenine and increased levodopa and acetylcholine chloride in SD mice (Figure 5d). Administration of 1× EC also significantly increased picolinic acid and histamine levels, while 2× EC had little effect on these two neurotransmitters in SD mice (Figure 5d).

### 3.6. Effect of EC on Serum Metabolite Profiles in SD Mice

**Figure 6:**
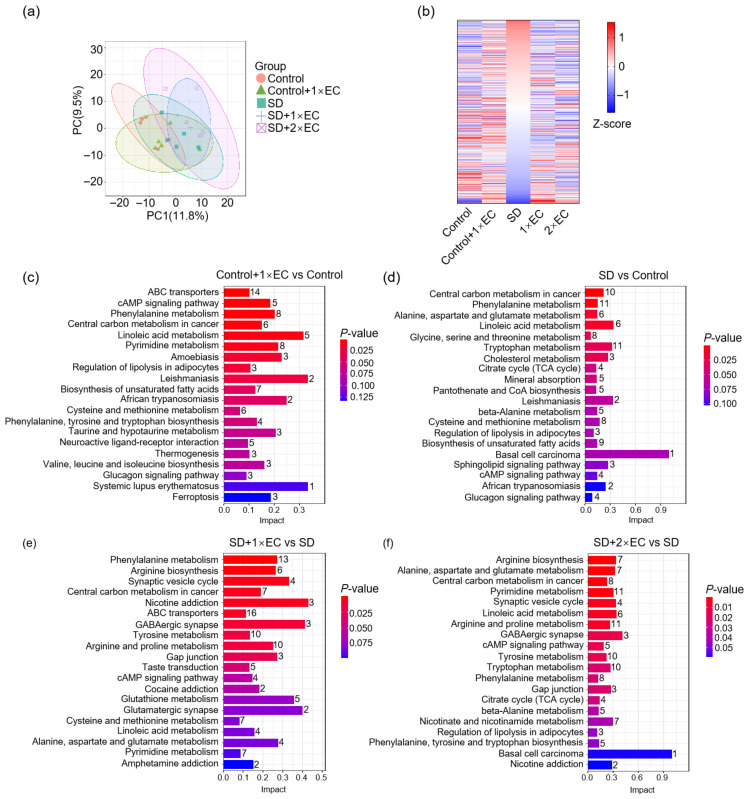
EC administration altered serum metabolomics of SD mice. (**a**) PCA of serum metabolomics by untargeted metabolomics. (**b**) Heatmap of serum metabolites in SD mice, the metabolites were plotted by the highest Z-score in the SD group. (**c**) KEGG pathways enriched in the Control + 1× EC group compared to the Control group. (**d**) KEGG pathways enriched in the SD group compared to the Control group. (**e**) KEGG pathways enriched in the SD + 1× EC group compared to the SD group. (**f**) KEGG pathways enriched in the SD + 2× EC group compared to the SD group.

Finally, untargeted metabolomics analysis of the serum was performed to determine whether the effect of EC on SD depends on specific bioactives or metabolites. Similarly to the results of neurotransmitters, PCA showed slight separation among different groups (Figure 6a). The heatmap of all detected metabolites indicated that EC treatment or SD resulted in a marked alteration in the levels of certain metabolites, and the three EC-treated groups also shared some common changes (Figure 6b). Next, the KEGG pathway was used to determine the pathways involved in the metabolite changes between the control + 1× EC group and the control group, the SD group and the control group, and the SD + 1× EC group or SD + 2× EC group and the SD group (Figure 6c–f). Firstly, there were several common pathways that were also enriched in the kinetic study at T30, such as central carbon metabolism in cancer; linoleic acid metabolism; regulation of lipolysis in adipocytes; phenylalanine, tyrosine, and tryptophan biosynthesis; neuroactive ligand–receptor interaction; valine, leucine, and isoleucine biosynthesis; and glucagon signaling pathway (Figure 6c). Secondly, SD and EC treatment resulted in a marked change in the top 20 enriched pathways, and the common pathways related to these three groups were central carbon metabolism in cancer; phenylalanine metabolism; alanine, aspartate, and glutamate metabolism; linoleic acid metabolism; and cAMP signaling pathway (Figure 6d–f), suggesting that the effect of EC on SD might be related to carbohydrate and amino acid metabolism, which is consistent with the nature of EC. In addition, the treatment with EC significantly enriched the synaptic vesicle cycle, GABAergic synapse, and tyrosine metabolism pathways (Figure 6d–f), suggesting that EC may enhance synaptic transmission, restore inhibitory–excitatory balance, and modulate dopaminergic signaling, thereby potentially counteracting SD-induced neural dysregulation.

### 3.7. EC-Induced Changes in Specific Active Substances in Serum Metabolites of SD Mice

**Figure 7:**
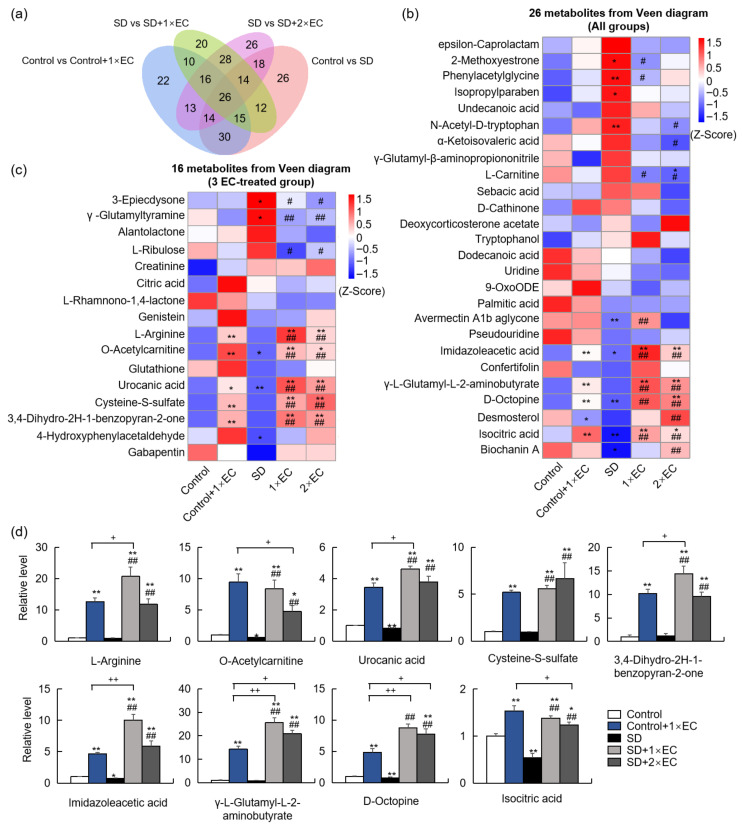
EC and SD altered several common serum metabolites. (**a**) Venn diagram of the number of metabolites altered by SD or EC. The number of metabolites was determined by fold-change between the two groups (Control vs. Control + 1× EC, Control vs. SD, SD vs. SD + 1× EC, and SD vs. SD + 2× EC) over 1.5-fold or less than 0.5-fold. (**b**) Heatmap of 16 common metabolites in 3 EC-treated groups. (**c**) Heatmap of 26 common metabolites in all groups. (**d**) The relative levels of 9 metabolites commonly increased under the Control and SD conditions. * *p* < 0.05, and ** *p* < 0.01 vs. the Control group, # *p* < 0.05, and ## *p* < 0.01 vs. the SD group. ^+^ *p* < 0.05, and ^++^ *p* < 0.01 SD + 1× EC and SD + 2× EC vs. Control + 1× EC.

A Venn diagram was used to identify the number of common metabolites found in different groups to find the specific metabolite changes after SD and EC treatment. A total of 42 common metabolites were found in the three EC-treated groups, and 26 common metabolites were found in all groups (Figure 7a). The change in these 42 metabolites was further visualized using a heatmap (Figure 7b,c). SD caused a significant increase in several metabolites, such as 3-epiecdysone, γ-glutamyltyramine, 2-methoxyestrone, phenylacetylglycine, isopropylparaben, and N-acetyl-D-tryptophan, and a marked decrease in several metabolites, including urocanic acid, 4-hydroxyphenylacetaldehyde, avermectin A1b aglycone, imidazoleacetic acid, D-octopine, isocitric acid, and biochanin A, which were greatly reversed by EC treatment (Figure 7b,c). EC treatment under the control and SD conditions resulted in a significant increase in some common metabolites, and these metabolites were L-arginine, O-acetylcarnitine, urocanic acid, cysteine-S-sulfate, 3,4-dihydro-2h-1-benzopyran-2-one, imidazolelacetic acid, γ-L-glutamyl-L-2-amino-butyrate, D-octopine, and isocitric acid (Figure 7b,c). Therefore, these increased metabolites might be biomarkers of EC supplementation.

To better understand the effect of EC on the serum metabolites, the relative levels of nine common metabolites were analyzed using bar graphs (Figure 7d). Consistent with the Z-score-derived heatmap, the relative levels of these metabolites were all significantly increased by EC under the control or SD condition (Figure 7d). In addition, the levels of L-arginine, urocanic acid, 3,4-dihydro-2H-1-benzopyran-2-one, imidazoleacetic acid, γ-L-glutamyl-L-2-aminobutyrate, and D-octopine in the SD + 1× EC group were higher than those of the control + 1× EC group, and the effect of 1× EC on these metabolites tended to be stronger than 2× EC group (Figure 7d). These results also suggested that 1× EC was sufficient to ameliorate the abnormal metabolite changes related to SD. Among them, L-arginine, γ-L-glutamyl-L-2-aminobutyrate, and 3,4-dihydro-2H-1-benzopyran-2-one showed the most significant changes. Therefore, these three metabolites may serve as potential biomarkers for the effects of EC.

### 3.8. Correlation Analysis of Differential Metabolites and SD-Related Genes

**Figure 8:**
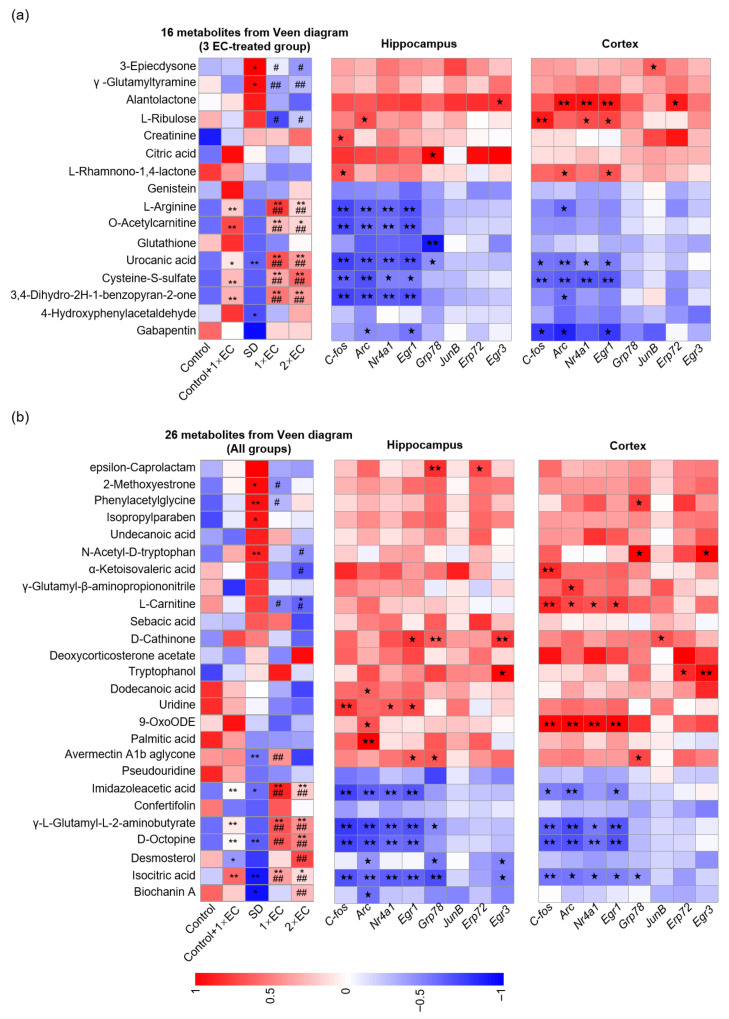
EC-altered metabolites were correlated with SD-related gene expression. (**a**) Correlation analysis between 16 common metabolites in 3 EC-treated groups and SD-related gene expression in the hippocampus and cortex. (**b**) Correlation analysis between 26 common metabolites in all groups and SD-related gene expression in the hippocampus and cortex. The correlation analysis was performed using the data from the Control, SD, SD + 1× EC, and SD + 2× EC groups. * *p* < 0.05, and ** *p* < 0.01 vs. the Control group, # *p* < 0.05, and ## *p* < 0.01 vs. the SD group. One pentacle (*p* < 0.05) and two pentacles (*p* < 0.01) mean significant correlations.

Next, a correlation analysis was performed between the common metabolites and the SD-related gene expression (Figure 8). Overall, the metabolites that were increased in the SD group were positively correlated with the expression of immediate early genes (IEGs) in the hippocampus and cortex, while the metabolites that were decreased in the SD group and increased by EC were negatively correlated with the expression of these genes (Figure 8). Specifically, alantolactone, L-ribulose, citric acid, L-carnitine, D-cathinone, uridine, and 9-OxoODE showed a strong positive correlation with the expression of IEGs (Figure 8). Importantly, the nine metabolites that were commonly increased by EC under the control and SD conditions—L-arginine, O-acetylcarnitine, urocanic acid, cysteine-S-sulfate, 3,4-dihydro-2h-1-benzopyran-2-one, imidazolelacetic acid, γ-L-glutamyl-L-2-amino-butyrate, D-octopine, and isocitric acid—were all negatively correlated with the expression of C-fos, activity-regulated cytoskeleton-associated protein (*Arc*), nuclear receptor (*Nr4a1*), and early growth response 1 (*Egr1*) (Figure 8), suggesting a high possibility that these metabolites not only act as biomarkers of EC, but could affect certain pathways in the brain.

## 4. Discussion

EC is a complex that contains bioactive peptides and amino acids [38]. After digestion and absorption, these ingredients can cause changes in relevant active metabolites in the blood and brain. For example, branched-chain amino acids (BCAAs, including valine, leucine, and isoleucine) undergo catabolism mediated by branched-chain aminotransferase isoenzymes and branched-chain α-ketoacid dehydrogenase [16]. Additionally, carnosine (a key bioactive constituent of EC) is hydrolyzed by serum carnosinase into L-histidine and β-alanine [39]. Thus, the ameliorative effect of EC on SD may involve changes in multiple active metabolites [24,40]. In this study, we observed significantly elevated levels of myristoleic acid, L-tyrosine, and trans-cinnamoyl beta-D-glucose in serum. Myristoleic acid is widely present in eukaryotic organisms and animal adipose tissues [41,42]. A previous study showed that EC increased lipoprotein lipase activity in fat tissues and improved blood lipid metabolism in stressed mice [43]. Therefore, the increase in myristoleic acid may reflect EC-mediated modulation of lipid metabolism. L-tyrosine is an amino acid abundant in chicken meat and extracts, which serves as a precursor for catecholamine neurotransmitters. It is converted into dihydroxyphenylalanine (DOPA), then dopamine, and ultimately norepinephrine and epinephrine, all of which play roles in emotion regulation and stress response [44,45]. Notably, L-tyrosine is converted to dopamine by tyro sine hydroxylase (TH), a circadian-regulated enzyme [46], suggesting L-tyrosine may influence dopamine production in a time-dependent manner. The KEGG results identified another active metabolite, trans-cinnamoyl beta-D-glucosid, derived from cinnamic acid, which is only found in plants. A possible reason for this might have been that the padding of the mouse cage was corncob, not wood shavings, in our animal facility. Sometimes, the mice will consume the corncob, and the trans-cinnamic acid is metabolized into trans-cinnamoyl-O-glucoside.

Unlike the changes in metabolites in serum, mainly concentrated at 15 and 30 min, we found that metabolic changes in the brain were most significant at 30 min, with marked differences in the types of metabolites compared to the serum. According to reports, small molecules enter the bloodstream through liver metabolism after digestion in the intestine and then selectively enter the brain through the BBB, thereby exerting a protective effect on the CNS [47]. Therefore, one of the main reasons for the differences in brain and serum metabolites might be the selective differences in the BBB. In addition, one possible factor affecting the brain metabolites in the control group at T15 was the acute stress caused by the gavage and handling of other mice in the same cage. Overall, the variation in metabolites in the control group was much less obvious than in the EC-treated groups. Gentisic acid is a redox-active quinonoid acetylsalicylic acid metabolite found in all organisms and is used as an adjunct for the treatment of mental illness [48]. Furthermore, gentisic acid has been reported to have strong free radical scavenging activity, which inhibits the formation of cholesterol ester hydroperoxides [49]. However, Kennedy et al. demonstrated that stress-induced fatigue is associated with increased lipid peroxidation [50]. In the present study, we observed increased levels of gentisic acid in the brain following EC supplementation, suggesting gentisic acid may represent a bioactive component of EC, potentially contributing to the inhibition of lipid peroxidation.

Neurotransmitters and their metabolites in the CNS play an important role in stress relief, mood regulation, and hypnosis [51]. 5-HT is one of the first neurotransmitters shown to be related to the sleep–wake cycle [52]. Oikonomou et al. found that when the dorsal raphe nucleus (DRN) of mice or zebrafish no longer produced 5-HT, the sleep time of these animals was significantly reduced, indicating that 5-HT is essential for sleep [53]. In addition, 5-HT can be further converted into melatonin, thereby regulating the sleep cycle [54]. Previous studies have shown that the nutritional components of EC, carnosine and anserine, are precursors of histamine synthesis [38]. Histamine can lower the levels of substances related to sleep disorders and stress, such as cortisol, by regulating the levels of 5-HT, thereby improving sleep quality [55,56]. Consistent with these findings, we found that the serum levels of 5-HT significantly decreased during SD, and EC tended to reverse this trend. In addition, 5-hydroxytryptop (5-HTP), as a precursor of serotonin, easily crosses the BBB and becomes serotonin under the action of 5-hydroxytryptop decarboxylase (5-HTPDC) [57]. We found that EC elevated serum 5-HTP levels in SD mice, suggesting a potential role in modulating serotonin synthesis, which is implicated in sleep regulation. Some studies previously reported that the administration of EC was able to increase the levels of 5-HT or its related compound, i.e., 5-hydroxyindole acetic acid (5-HIAA), in different tissues such as the hippocampus, cortex, plasma, and cerebrospinal fluid [58,59], which was also verified in our study. Particularly in a restraint stress model, EC administration could also recover the changed levels of 5-HT in the brain or plasma, strongly suggesting that EC exerts its benefits through modulation of tryptophan hydroxylase activity [59].

EC treatment not only significantly enriched the pathways of amino acid metabolism but also significantly enriched the pathways of the synaptic vesicle cycle, GABAergic synapse, and tyrosine metabolism. GABA is the primary inhibitory neurotransmitter in the central nervous system, and it exerts its effects through GABAergic synapses to modulate neuronal excitability and synaptic transmission [60]. The metabolism of tyrosine, a precursor to neurotransmitters such as dopamine, adrenaline, and noradrenaline, is crucial for the function of the nervous system and the regulation of mood [61]. These results propose a hypothesis that EC may influence the synthesis of neurotransmitters by modulating GABAergic synaptic transmission and tyrosine metabolism, and relevant experiments to further validate this hypothesis need to be conducted in future studies. Moreover, GABA is formed by the decarboxylation of glutamate via glutamate decarboxylase in GABAergic neurons, which plays a key role in controlling the sleep–wake balance [62]. Many studies have shown that GABA has a sleep-promoting effect. For example, Byun et al. tested 40 insomniacs treated with GABA for 4 weeks and found that their sleep latency was shortened and their sleep efficiency increased after treatment [63]. Acetylcholine chloride (Ach) is recognized as a participant in rapid eye movement (REM) sleep. Enhancing the release of Ach in the pontine reticular structure can enhance REM sleep in cats, rats, and mice [64]. In addition, Magill et al. reported that supplementing tyrosine after SD improved working memory, reasoning, and alertness in healthy young men [65]. Consistent with these findings, our results showed decreased serum levels of these neurotransmitters after SD, which were partly reversed by EC, highlighting the protective role of EC during SD. SD alone was slightly affected, but SD with EC treatment caused a more obvious change in some neurotransmitters, such as kynurenic acid and kynurenine. A possible reason for this might be that SD causes some physiological changes in mice, such as fatigue and stress, and EC treatment may ameliorate these changes and further impact the level of neurotransmitters. On the other hand, in addition to generating 5-HTP, another metabolic pathway for tryptophan is the production of kynurenic acid and kynurenine, which may adversely affect neural function [66]. EC treatment significantly reduced the levels of kynurenic acid and kynurenine, suggesting that it may shift tryptophan metabolism towards the serotonergic pathway instead of the kynurenine pathway in SD mice.

Enrichment analysis of metabolic pathways revealed that carbon metabolism, amino acid biology, and cAMP signaling pathways were significantly enriched in SD mice following EC ingestion. Amino acid metabolism plays an important role in redox balance, energy regulation, biosynthetic support, and homeostasis maintenance [67]. The disruption of phenylalanine metabolism in phenylketonuria can lead to deficiencies in the sleep regulators dopamine, noradrenaline, and 5-HT, thereby affecting sleep-related processes [68]. Previous studies have shown that EC contains essential amino acids, such as threonine, valine, methionine, isoleucine, leucine, phenylalanine, lysine, and tryptophan [25], and a significant proportion of hydrolyzed amino acids, such as glycine, alanine, glutamate, and aspartate [69]. Therefore, EC or its biological components may improve mental fatigue through some amino acid metabolism pathways after being metabolized and absorbed in vivo. Research has shown that cAMP mediates different cellular responses to extracellular signals, cellular stress responses, and other signaling pathways [70]. SD reduces cAMP signaling in the hippocampus of mice, leading to AMPK receptor phosphorylation and a reduction in CREB-mediated gene transcription [71]. In the present study, we observed elevated levels of L-arginine following EC supplementation, which serves as a precursor for nitric oxide (NO) synthesis. This is mechanistically significant because NO activates soluble guanylate cyclase (sGC) to produce cGMP, which in turn modulates cAMP levels through phosphodiesterase 3 (PDE3) inhibition [24,72]. These interconnected pathways suggest that the elevated L-arginine may potentiate cAMP signaling via cGMP, pointing to a potential mechanism through which EC could restore cAMP homeostasis and ameliorate SD-induced deficits. However, this proposed mechanism remains to be rigorously tested. To fill this gap, further experimental validation of the involvement of the cAMP signaling pathway will be conducted to substantiate these findings. In addition, we found that EC supplementation resulted in a significant increase in urocanic acid under SD conditions. Urocanic acid is an intermediate product in histidine metabolism, and both arginine and histidine are abundant in chicken essence [73]. It has been reported that enhanced functions associated with L-arginine biosynthesis improve rodent memory impairment [74]. In a recent study, Wang et al. demonstrated that urocanic acid promotes the urocanic acid–glutamate metabolism pathway and enhances glutamatergic transmission, thereby promoting memory recovery in mice [75]. These results suggest that L-arginine and urocanic acid may be partial specific biomarkers of EC regulating sleep-related neural functions. However, further experimental validation is indeed required to elucidate their specific roles in mediating the effects of EC, which is one of the limitations of our study.

SD influences gene expression through diverse mechanisms, including epigenetic modification, transcriptional regulation, and mRNA processing [76]. Through hippocampal transcriptome dynamics after acute SD, 1146 genes were found to be significantly dysregulated after SD in mice [20]. Among these, immediate early genes (IEGs) are rapidly and transiently activated by cellular stimuli, which regulate the interaction between neurons and key brain functions [77]. Terao et al. found that IEG expression was strongly influenced and increased during a 6 h SD period [78], consistent with other studies showing SD upregulated the expression of several IEGs (e.g., *C-fos*, *Egr1*, *Arc*, *Nr4a*) and their alternative splice variants [79]. These IEGs encode proteins that regulate diverse cellular processes, ultimately influencing neuronal plasticity and brain function: *Arc* modulates genes involved in plasticity, mitogenesis, and differentiation (e.g., synapsins I/II, synaptobrevin II) [80], while *C-fos* acts as a general transcription factor [79]. In this study, we found that SD significantly increased the expression of IEGs in the cortex and hippocampus, which was downregulated by EC. These results suggested that EC may downregulate the expression of IEGs, further studies are needed to determine whether this downregulation leads to altered levels of downstream molecules, such as transcription factors, growth factors, or enzymes, to alleviate the effects of SD. Concurrent with these transcriptional changes in the hippocampus and cortex, SD also significantly altered the expression of key enzymes governing melatonin synthesis in the pineal gland, which is a master regulator of circadian rhythms. The pineal gland converts 5-HT to melatonin, a process critically dependent on the rate-limiting enzyme *Tph2* for serotonin production and *Aanat* for melatonin synthesis [81]. Specifically, we found that SD downregulated the expression of *Tph2* and *Aanat*, thereby likely impairing melatonin production [81,82]. Conversely, SD upregulated the expression of *Mao-b*, which degrades monoamines, including serotonin, and thus further reduces substrate availability for melatonin synthesis [83]. Collectively, the downregulation of melatonin biosynthetic enzymes (*Tph2*, *Aanat*) and upregulation of the catabolic enzyme (*Mao-b*) suggest a potential mechanism by which SD disrupts circadian rhythmicity. Notably, EC supplementation effectively reversed these SD-induced changes in pineal enzyme expression, indicating a protective role of EC supplementation in preserving pineal function and melatonin-driven circadian regulation against SD-induced disruption.

We performed a correlation analysis of 42 common metabolites with SD-related genes and found that the increased metabolites after EC intake were negatively correlated with the expression of SD genes. This further validated the potential of these metabolites as biomarkers of EC. However, compared to the previous serum kinetic study, SD caused significant changes in different serum metabolites in mice, such as 2-Methoxyestrone, phenylacetylglycine, isopropylparaben, N-acetyl-D-tryptophan, avermectin A1b aglycone, imidazoleacetic acid, and isocitric acid. A study on 12 healthy male subjects found that SD caused disruptions in the bioclock and led to changes in the metabolic composition of the blood [35]. In addition, a single metabolomics study attempting to identify the pathways activated by waking in mice also showed that relatively few metabolite pathways were affected by 6 h of SD, including glycolysis and lipid metabolism [84]. Therefore, the difference in metabolites between the serum kinetic study and the SD experiments may have been caused by the time difference of 6 h and model differences.

The identified metabolites may serve as potential biomarkers mediating the effects of EC on SD. Specifically, L-arginine serves as an essential amino acid mediating multiple physiological processes, including NO production and neurotransmitter synthesis. Our findings demonstrate that EC treatment significantly elevates serum levels of L-arginine, γ-L-glutamyl-L-2-aminobutyrate (a glutamate metabolite regulating neurotransmitter balance), and 3,4-dihydro-2H-1-benzopyran-2-one, suggesting that these metabolites may function as potential biomarkers of the effects of EC on SD. While the sleep-promoting properties of L-arginine through NO-dependent mechanisms are established [85], and γ-L-glutamyl-L-2-aminobutyrate may modulate glutamatergic tone, the neurobiological significance of 3,4-dihydro-2H-1-benzopyran-2-one requires further investigation. Notably, EC is widely used as a nutritional supplement in daily life [86]. In previous reports on physiological function, EC demonstrated biological activity in combating mental fatigue by activating the histaminergic system and regulating plasma lactate and ammonia levels [87]. However, no studies have reported on changes in specific bioactivities after ingestion of EC. To our knowledge, this is the first study to report an effect of EC on regulating sleep disorders in an SD mouse model, which can be achieved by altering specific biomarkers in the serum. However, the specific pathways through which EC regulates SD have not been fully studied. For example, the upstream mechanism activating cAMP signaling has not been experimentally elucidated. This is also one of the limitations of this study. In future research, we will conduct in vitro and in vivo experiments to further verify the biological functions of these metabolites. Moreover, the mechanisms of the effect of EC on SD and the most active compounds in EC that are responsible for its effect remain largely unknown and require further investigation.

## 5. Conclusions

In summary, we identified significant changes in biomarkers in the serum and brain of mice after EC administration and metabolomic changes after EC intake during SD, and these changes were time-dependent. Under acute SD conditions, EC may regulate sleep-related neural functions by upregulating the blood 5-HT content, alleviating abnormal neurotransmitters, and altering some serum biomarkers. These results provide clues for further understanding the multiple roles of EC.

## Data Availability

The original contributions presented in this study are included in the article and Supplementary Materials. Further inquiries can be directed to the corresponding author.

## Supplementary Materials

The following supporting information can be downloaded at https://www.mdpi.com/article/10.3390/metabo15090577/s1, Table S1: Primer sequence for RT-qPCR; Figure S1: KEGG pathway analysis of serum metabolites in 1× EC and 2× EC group after 15 and 30 min administration; Figure S2: Line graphs of serum metabolites significantly affected by EC; Figure S3: KEGG pathway analysis of brain metabolites in 1× EC and 2× EC group after 15 and 30 min of administration; Figure S4: Line graphs of brain metabolites significantly affected by EC; Figure S5: mRNA expression of SD-related markers in the cortex, hippocampus, and pineal gland.

## Abbreviations

EC	Essence of chicken
SD	Sleep deprivation
GC/MS	Gas chromatography coupled with mass spectrometry
CNS	Central nervous system
BBB	Blood–brain barrier
LC-MS	Liquid chromatography–mass spectrometry
BW	Body weight
ELISA	Enzyme-linked immunoassay
5-HT	5-hydroxytryptamine
KEGG	Kyoto Encyclopedia of Genes and Genomes
HMDB	Human Metabolome Database
SEM	Standard error of the mean
ANOVA	One-way analysis of variance
PCA	Principal coordinate analysis
ABC	ATP-binding cassette
IEGs	Immediate early genes
*Arc*	Activity-regulated cytoskeleton-associated protein
*Nr4a1*	Nuclear receptor
*Egr1*	Early growth response 1
*Tph2*	Tryptophan hydroxylase 2
*Aanat*	Arylalkylamine N-acetyltransferase
*Mao-b*	Monoamine oxidase B
BCAA	Branched-chain amino acid
DOPA	Dihydroxyphenylalanine
DRN	Dorsal raphe nucleus
5-HTP	5-hydroxytryptop
5-HTPDC	5-hydroxyindole acetic acid
Ach	Acetylcholine chloride
No	Nitric oxide
REM	Rapid eye movement
