# Supplementary material for: Essence of Chicken Supplementation Alters Brain and Blood Metabolite Signatures in Sleep-Deprived Mice
Source: Metabolites. 2025 Aug 29;15(9):577. doi: 10.3390/metabo15090577 (PMC12471716; doi:10.3390/metabo15090577)
Supplement: Supplementary file 1 [file metabolites-15-00577-s001.zip › metabolites-3765894-supplementary.pdf]

Article

Essence of chicken supplementation altered brain and blood metabolite signatures in sleep-deprived mice

Supplementary Figures:

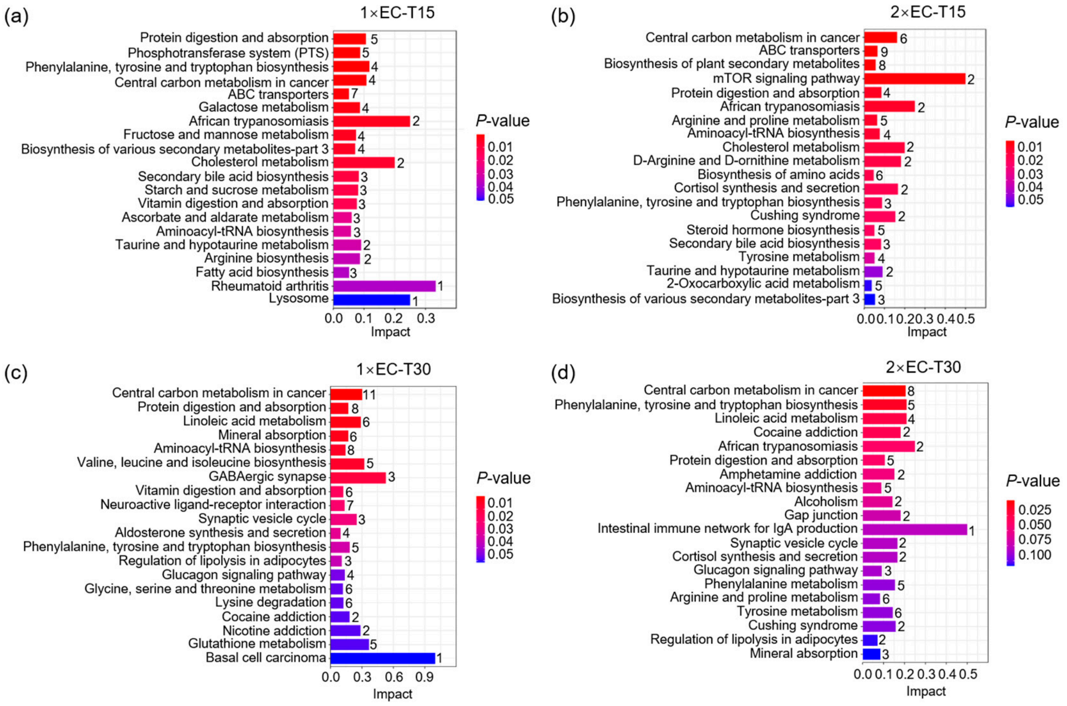

Figure S1. KEGG pathway analysis of serum metabolites in 1×EC and 2×EC group after 15 and 30 min administration. (**a**) Pathways enriched in the 1×EC group at T15. (**b**) Pathways enriched in the 2×EC group at T15. (**c**) Pathways enriched in the 1×EC group at T30. (**d**) Pathways enriched in the 2×EC group at T30. The horizontal axis represents the proportion of the number of differential metabolites under this pathway to the total number of all differential metabolites; The vertical axis represents the path name; The *P*-value represents the significant degree of enrichment, and the darker the color, the smaller the *P*-value and the higher the enrichment degree.

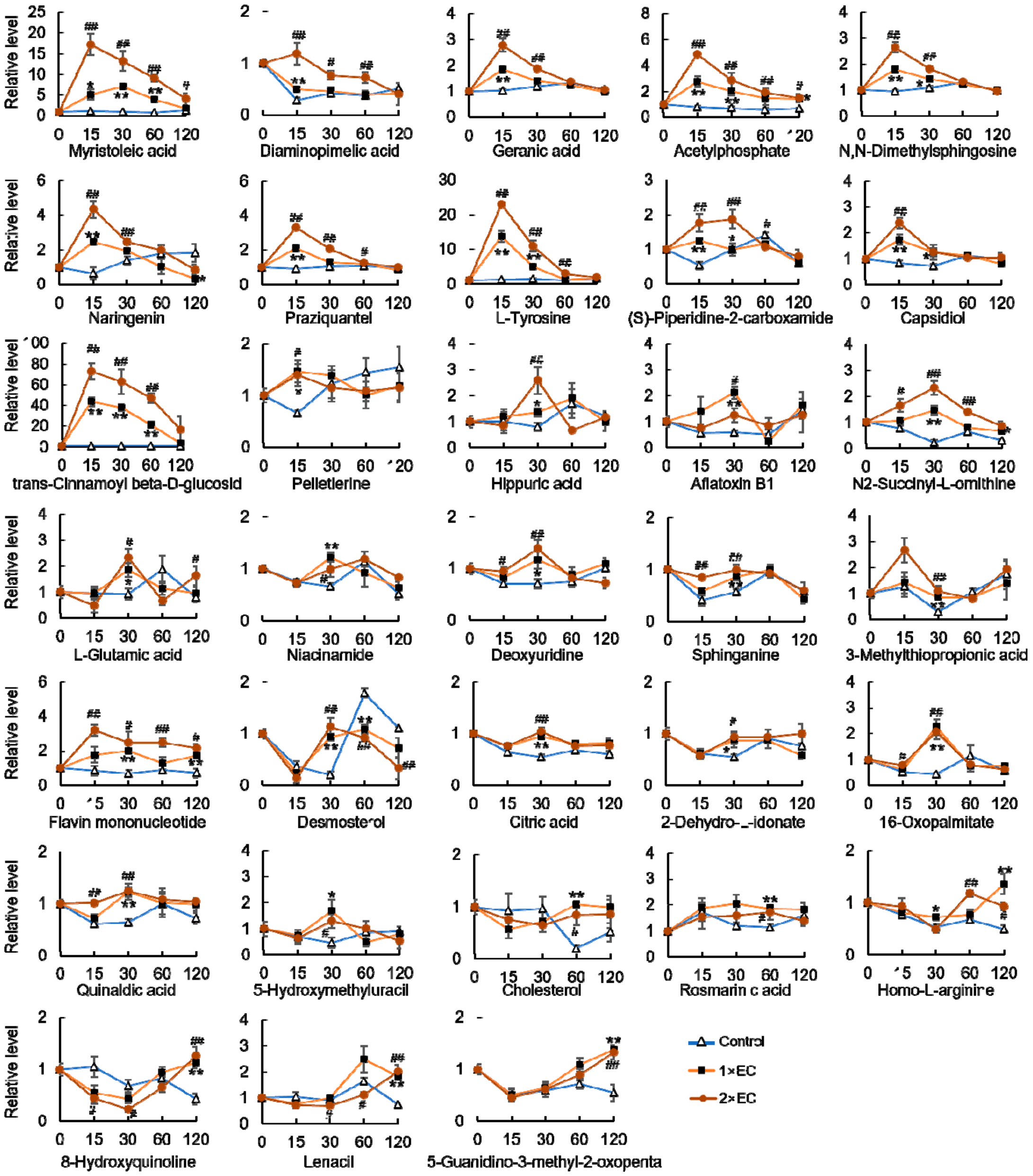

Figure S2. Line graphs of serum metabolites significantly affected by EC. 33 metabolites significantly increased by EC from Figure 3 (total 50 metabolites), and the abnormal change metabolites were removed. *$P < 0.05$, **$P < 0.01$, 1×EC vs. the Control group, #$P < 0.05$, ##$P < 0.01$, 2×EC vs. the Control group.

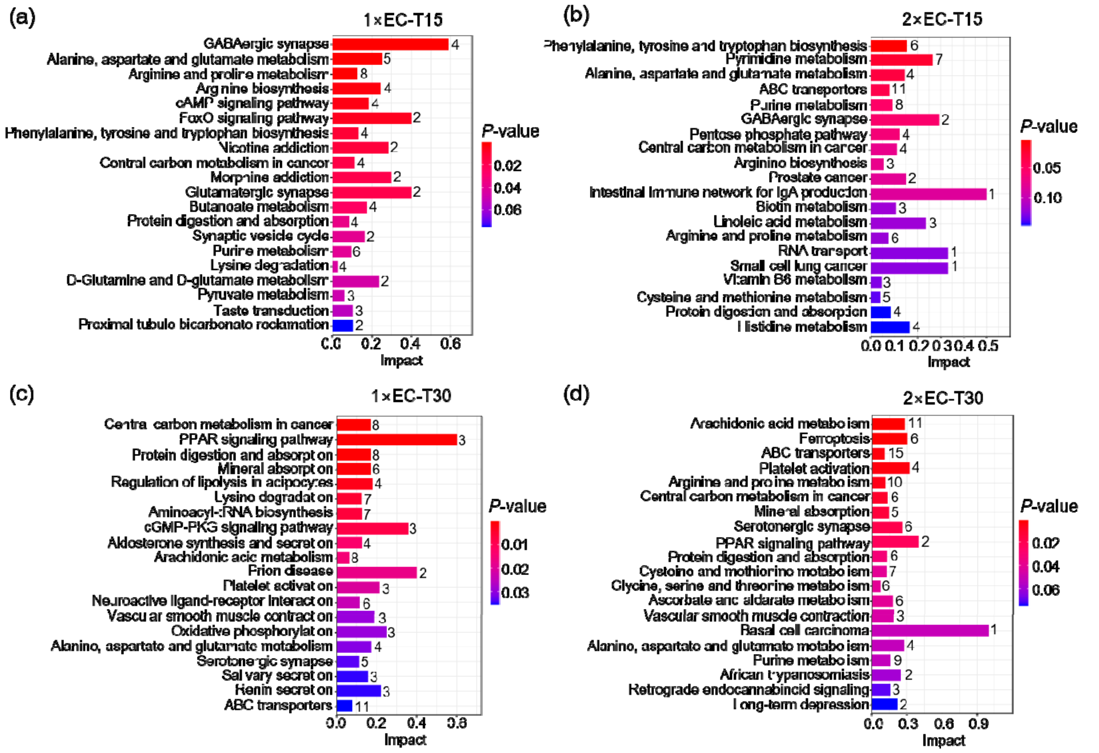

Figure S3. KEGG pathway analysis of brain metabolites in 1×EC and 2×EC group after 15 and 30 min of administration. (**a**) Pathways enriched in the 1×EC group at T15. (**b**) Pathways enriched in the 2×EC group at T15. (**c**) Pathways enriched in the1×EC group at T30. (**d**) Pathways enriched in the 2×EC group at T30. The horizontal axis represents the proportion of the number of differential metabolites under this pathway to the total number of all differential metabolites; The vertical axis represents the path name; The *P*-value represents the significant degree of enrichment, and the darker the color, the smaller the *P*-value and the higher the enrichment degree.

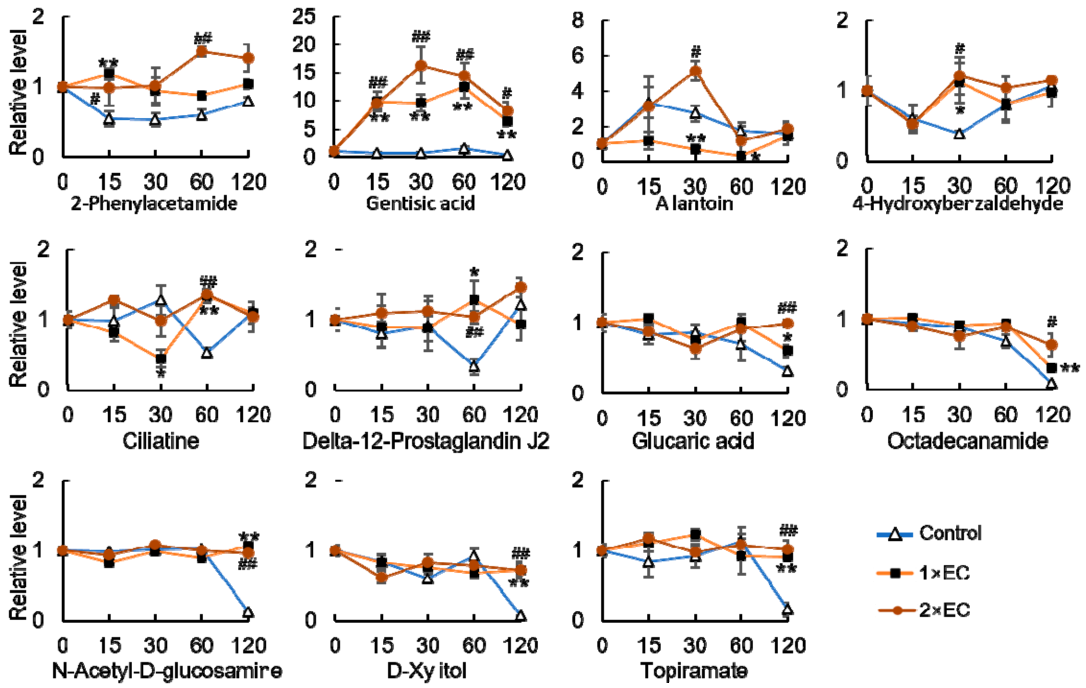

Figure S4. Line graphs of brain metabolites significantly affected by EC. 11 metabolites significantly increased by EC from Figure 7 (total 15 metabolites), and the abnormal change metabolites were removed. $^{*}P < 0.05$, $^{**}P < 0.01$, 1×EC vs. the Control group, $^{\#}P < 0.05$, $^{\#\#}P < 0.01$, 2×EC vs. the Control group.

Figure S5

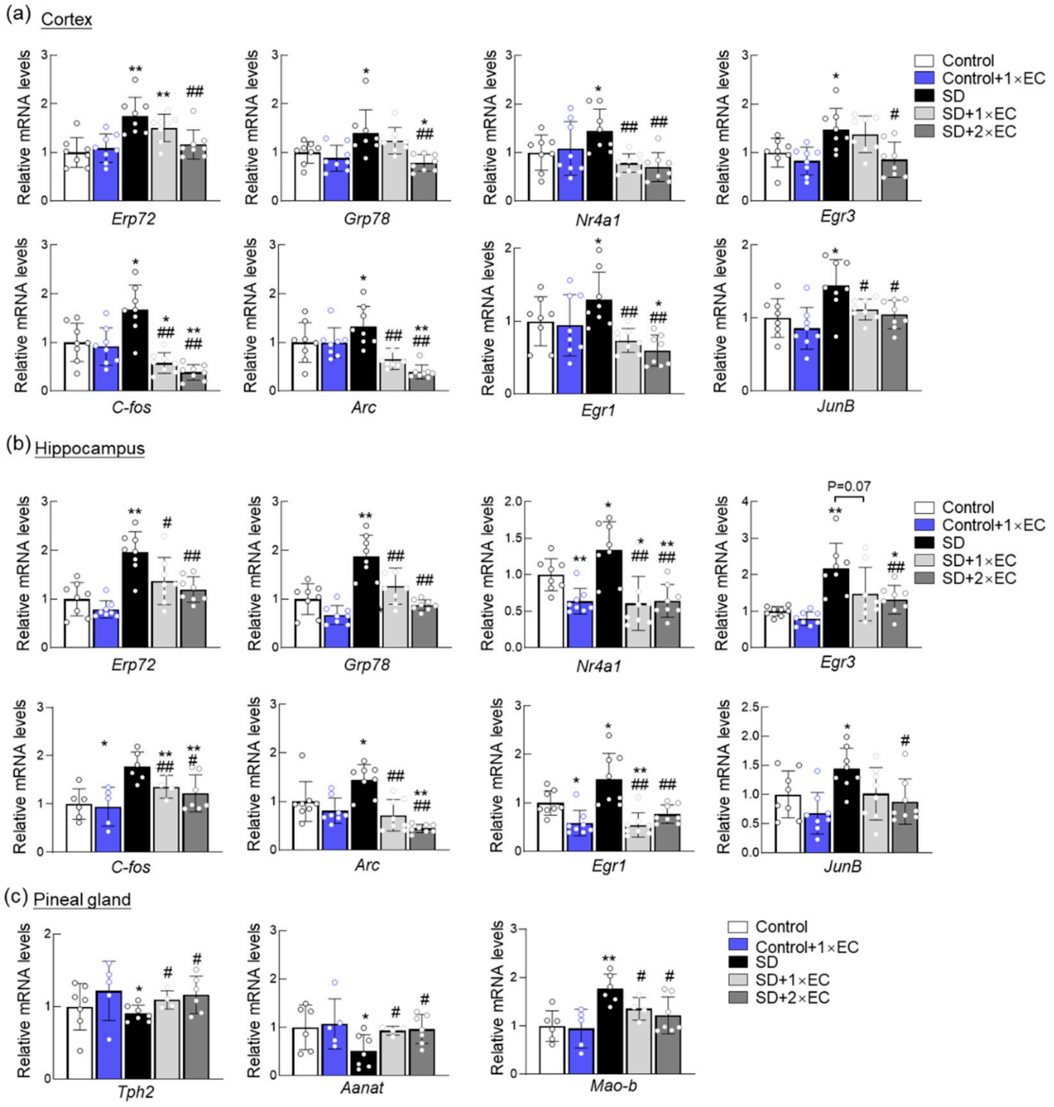

Figure S5. mRNA expression of SD-related markers in the cortex, hippocampus, and pineal gland. (**a**) mRNA expression of SD-related markers in the cortex. (**b**) mRNA expression of SD-related markers in the hippocampus. (c) mRNA expression of SD-related circadian rhythms markers in the pineal gland. $^{*}P < 0.05$, $^{**}P < 0.01$ vs. the Control group, $^{\#}P < 0.05$, $^{\#\#}P < 0.01$ vs. the SD group.

Supplementary Tables：

Table S1. Primer sequence for RT-qPCR

Name	Primer 5′ to 3′ Forward	Primer 5′ to 3′Reverse
mERp72	CATCGCCAAGATGGATGCTA	ATAGATGGTAGGGAAGCCCTCC
mGRP78	CACGTCCAACCCCGAGAA	ATTCCAAGTGCGTCCGATG
mNr4a1	GAGTTCGGCAAGCCTACCAT	GTGTACCCGTCCATGAAGGTG
mEgr3	TTGCCTGACAATCTGTACCCC	TAATGGGCTACCGAGTCGCT
mC-fos	CGGGTTTCAACGCCGACTA	TGGCACTAGAGACGGACAGAT
mArc	GGTAAGTGCCGAGCTGAGATG	CGACCTGTGCAACCCTTTC
mEgr1	TCGGCTCCTTTCCTCACTCA	CTCATAGGGTTGTTCGCTCGG
mJunB	TCACGACGACTCTTACGCAG	CCTTGAGACCCCGATAGGGA
mβ-actin	CTGAGAGGGAAATCGTGCGT	CCACAGGATTCCATACCCAAGA
Tph2	CAGGGTTACTTTCCTCCATCG	AGCAGGTTGTCTTCGGGTCA
Aanat	TGAGCGGGAAGCCTTTATCTC	CTCCTGAGTAAGTCTCTCCTTGT
Mao-b	TGGGAAGATTCCAGAGGATG	GCTGACAAGATGGTGGTCAA
